# Plasma cytokines and risk of coronary heart disease in the PROCARDIS study

**DOI:** 10.1136/openhrt-2018-000807

**Published:** 2018-04-25

**Authors:** Robert Clarke, Elsa Valdes-Marquez, Michael Hill, Joanne Gordon, Martin Farrall, Anders Hamsten, Hugh Watkins, Jemma C Hopewell

**Affiliations:** 1 Clinical Trial Service Unit and Epidemiological Studies Unit, Nuffield Department of Population Health, University of Oxford, Oxford, UK; 2 Radcliffe Department of Medicine, The Wellcome Trust Centre for Human Genetics, University of Oxford, Oxford, UK; 3 Cardiovascular Medicine Unit, Department of Medicine, Karolinska Institutet, Stockholm, Sweden

**Keywords:** cytokines, coronary heart disease

## Abstract

**Objective:**

The aims of the study were to examine the associations of plasma levels of five cytokines (interleukin (IL)-6, IL-5, interferon-gamma (IFN-γ), tumour necrosis factor-alpha (TNF-α) and IL-6 receptor (IL-6R)) and C reactive protein (CRP) with risk of coronary heart disease (CHD).

**Methods:**

In a case–control study of 931 CHD cases and 974 controls, logistic regression was used to estimate the OR and 95% CI of CHD for extreme thirds of biomarkers after adjustment for established risk factors. Sensitivity analyses were conducted in non-statin and in non-aspirin users.

**Results:**

Plasma levels of CRP were moderately correlated with IL-6 (r=0.45) in controls, but more weakly correlated with other cytokines. Likewise, all other cytokines were only weakly correlated with each other. After adjustment for established risk factors, the ORs (95% CI) for CHD comparing extreme thirds of cytokine levels (defined in controls) were 2.53 (1.86 to 3.43) for IL-6, 1.46 (1.11 to 1.93) for IL-5 and 1.46 (1.09 to 1.95) for IFN-γ, respectively. However, neither TNF-α, IL-6R nor CRP was significantly associated with CHD. After further adjustment for the associated cytokines, only IL-5 (1.34; 1.00 to 1.80) and IL-6 (2.39; 1.73 to 3.30) remained significantly associated with CHD. The risk associations of cytokines in non-users of statins or aspirin were comparable with the overall population.

**Conclusions:**

This study confirmed the importance of IL-6 as the most strongly associated cytokine with CHD risk, but also demonstrated novel and independent associations of IL-5 with CHD that warrant further investigation using larger panels of cytokines.

**Key questions**

**What is already known about this subject?**

- The recent results of the Canakinumab Anti-inflammatory Thrombosis Outcomes Study trial confirmed the independent relevance of inflammation in the aetiology of coronary heart disease (CHD) and demonstrated that inhibition of interleukin (IL)-1β to lower plasma levels of IL-6 reduced the risk of CHD.

**What does this study add?**

- The present study confirmed the importance of IL-6 as the most strongly associated cytokine with risk of CHD, but also demonstrated novel and independent associations of IL-5 with risk of CHD that warrant further investigation.

**How might this impact on clinical practice?**

- The results provide evidence to support the role of inflammation in the aetiology of CHD which could enhance our understanding of the biology of CHD and also lead to the discovery of novel treatments.

## Introduction

Inflammation has been implicated in the incidence and progression of coronary heart disease (CHD), but the role of the underlying inflammatory pathways in the pathogenesis of CHD is not fully understood.^1^ C reactive protein (CRP) is the most widely studied inflammatory marker for prediction of risk of CHD, but CRP is not causally related to CHD.^2^ CRP is produced by the liver in response to the action of cytokines which are the chemical messengers of the immune system secreted by activated macrophages and other immune and non-immune cells to control cell function.^3^ Established CHD risk factors (eg, smoking, hyperlipidaemia, hypertension and diabetes) trigger a cascade reaction whereby release of very low concentrations of one cytokine recruits inflammatory cells to trigger the production of other cytokines and amplifies a local inflammatory response.^3^

Circulating cytokines include interferon-gamma (IFN-γ) and tumour necrosis factor-alpha (TNF-α), which are released by T lymphocytes and trigger monocytes and macrophages to produce interleukin (IL)-1 and IL-6 that act on endothelial and smooth muscle cells in the arterial wall.^3^ Genetic studies provide strong support for a causal role of IL-6 for CHD.^4^ Recently, the Canakinumab Anti-inflammatory Thrombosis Outcomes Study (CANTOS) trial confirmed the reversibility of the excess risk associated with inflammation through inhibition of IL-1β for prevention of CHD.^5 6^ In the CANTOS trial, allocation of participants with a previous myocardial infarction (MI) and plasma levels of CRP >2 g/L to canakinumab that lowers plasma IL-6 levels (and CRP) resulted in a 15% reduction in cardiovascular disease events.^6^ The results of Mendelian randomisation (MR) studies and the CANTOS trial have both prompted interest in assessing associations of other cytokines with risk of CHD.^4 6^

Alternative cytokine pathways include IL-5, which is secreted by T helper 2 cells that act on eosinophils to release other proteins (eosinophil basic protein, neurotoxin, peroxidase and leukotrienes) that may influence atherosclerosis independent of the IL-1 and IL-6 pathways. Previous genetic studies reported associations of genetic variants encoding higher plasma levels of IL-5 with higher risk of CHD,^7^ but these associations have not been confirmed in subsequent large-scale genome-wide association studies of CHD^8–10^ or carotid atherosclerosis.^11^ Moreover, the advent of anti-IL-5 therapy (IL-5 antibodies and anti-IL-5 receptor antibodies) has also prompted interest in the study of IL-5 and risk of CHD.^12 13^

The aims of the present analyses of the PROCARDIS case–control study were: (1) to study the inter-relationships of cytokines with each other and (2) to assess the independent relevance for CHD of plasma levels of five cytokines (IL-6, IL-5, IFN*-*γ, TNF-α and IL-6R) and of CRP before and after adjustment for established CHD risk factors.

## Methods

### Study population

CHD cases were recruited from four European countries (UK, Italy, Sweden and Germany) between 2004 and 2008 into a retrospective case–control study of CHD.^14^ Cases had been previously diagnosed with CHD and also had a sibling who had been diagnosed with CHD before the age of 66 years. Ascertainment criteria for CHD cases were: (1) confirmed diagnosis of MI (based on standard ECG and enzyme criteria) or (2) acute coronary syndrome or stable angina cases that had a coronary revascularisation procedure (coronary artery bypass graft or coronary angioplasty) and a sibling with a diagnosis of MI or acute coronary syndrome before the age of 66 years. In addition, all cases with angina also had a history of coronary artery revascularisation. Cases were identified from disease registries of CHD events that typically occurred several years prior to blood collection. Controls were recruited from the same population as the cases (chiefly from spouses or siblings of spouses of CHD cases) and had no personal or sibling history of CHD before the age of 66 years. For each CHD case, it was planned to select one control of the same sex, country and age within 5 years of cases, using a frequency matching approach. In the UK, cases were identified with MI or unstable angina by the age of 66 years from hospital records used previously to recruit patients for large-scale trials of cholesterol-lowering therapy. Likewise, cases in Italy were identified through hospitals that had collaborated in the GISSI trials, in Sweden through existing registers of cases that had an MI at a young age and in Germany through the PROCAM and related databases. The study procedures were approved by the ethics committee of each participating country, and all participants provided written informed consent.

### Laboratory methods

Five cytokines were selected (IL-6, IL-5, IFN*-*γ, TNF-α and IL-6R) based on previous reports of associations with CHD and assay availability.^7–11^ Plasma cytokine levels were measured in duplicate on the same plate by a sandwich immunoassay with electrochemiluminescence detection using a Meso Scale Diagnostics platform (Gaithersburg, Maryland, USA) at the same laboratory in Oxford. The coefficients of variation (CVs) for individual assays (when measured once) were estimated using four quality control (QC) values per level on each plate (ie, involving a total of 220 QC values). These interassay CVs ranged from 9.6% to 10.9% for IL-6, 8.9% to 13.1% for IL-5, 16.5% to 33.0% for IFN*-*γ, 12.5%–16.5% for TNF-α and 13.9%–14.4% for IL6-R. The values for the cytokines presented in this report are geometric mean (95% CIs) levels of duplicate measurements of each cytokine.

### Statistical methods

Plasma levels of cytokines were measured on 1000 cases and 1000 controls, but valid assay results were only available on 931 (93%) cases and 974 (97%) controls. The distributions of the various cytokines were highly skewed, and consequently, all analyses were based on natural logarithm transformed values. Spearman correlation coefficients were calculated in controls to assess the correlations of cytokines with each other and with other plasma biomarkers (low-density lipoprotein cholesterol (LDL-C), high-density lipoprotein cholesterol, triglycerides, cystatin C, fibrinogen and CRP). Cytokine values were then grouped into thirds, based on control values, and logistic regression models were used to assess the association across thirds of each cytokine with CHD risk before and after adjustment for established CHD risk factors (age, sex, country, smoking status, history of hypertension, diabetes status, lipids and body mass index (BMI)). The ORs and their 95% CI for the top third relative to the bottom third of the cytokines studied were reported, with p values for trend across thirds. Sensitivity analyses were repeated in subsets in which both cytokine measures were within range (CV <20% and values within the measurable range for each assay). For cytokines that were significantly associated with CHD, models were also adjusted for all other cytokines associated with CHD. Additional sensitivity analyses examined the associations of cytokines with risk of CHD in subsets of participants who were non-users of statins or non-users of aspirin therapy. Empirical estimates of variance were used to account for familial clustering. P values <0.05 were considered statistically significant. Analyses were performed using SAS V.9.3, and figures were made using R V.2.14.1.

## Results

### Baseline characteristics

Selected characteristics of the 931 CHD cases and 974 controls with valid data on cytokines are shown in table 1. The mean (SD) age at diagnosis of MI, coronary artery bypass operation and coronary angioplasty was 54 (8) years, 56 (6) and 57 (7), respectively. The mean (SD) age at blood collection in all CHD cases was 63 (7) years and in controls was 61 (10). Hence, the mean (SD) interval between diagnosis and blood collection was 10 (6) years for MI, 7 (6) for coronary artery bypass operation and 5 (4) for coronary angioplasty. CHD cases had higher proportions of current smokers, hypertension and diabetes than controls as well as higher mean levels of BMI. Importantly, 67% of cases and 0% of controls reported current use of statins, and 75% of cases and 8% of controls reported current use of aspirin therapy at the time of blood collection. Consequently, plasma levels of LDL-C were lower in CHD cases than in controls. In contrast, the geometric mean CRP levels were higher in cases than in controls (2.10 versus 1.35 g/L), indicating that elevated levels of inflammation persisted for up to 10 years after the initial coronary event.

**Table 1:** Selected characteristics of CHD cases and controls

Mean (SD) or %*	Controls (n=974)	Cases † (n=931)
Baseline characteristics		
Age, years	60.9 (10.1)	62.9 (6.8)
Sex, female, %	299 (30.7)	289 (31.0)
Current smoker, %	190 (19.5)	392 (42.1)
Hypertension, %	222 (22.8)	482 (51.8)
Diabetes, %	15 (1.5)	149 (16.0)
Body mass index, kg/m^2^	26.7 (4.0)	28.5 (4.7)
Statin use, %	0 (0)	624 (67.0)
Aspirin use, %	74 (7.6)	702 (75.4)
Lipids and biomarkers of inflammation		
LDL-C, mmol/L	3.3 (0.8)	2.8 (0.8)
HDL-C, mmol/L	1.4 (0.4)	1.1 (0.3)
Triglycerides*, mmol/L	1.45 (1.40 to 1.50)	1.88 (1.81 to 1.94)
Fibrinogen, g/L	3.8 (0.9)	4.3 (1.0)
Cystatin C*, mg/L	0.76 (0.75 to 0.76)	0.85 (0.84 to 0.87)
CRP*, g/L	1.35 (1.26 to 1.45)	2.10 (1.95 to 2.26)
Cytokines		
IL-6*, pg/mL	0.61 (0.59 to 0.64)	1.03 (0.98 to 1.08)
IL-5*, pg/mL	0.17 (0.17 to 0.18)	0.21 (0.20 to 0.23)
IFN-γ*, pg/mL	1.03 (0.97 to 1.09)	1.16 (1.10 to 1.22)
TNF-α*, pg/mL	6.64 (6.45 to 6.82)	7.18 (6.95 to 7.42)
IL-6R*, pg/mL	818.94 (805.35 to 832.77)	817.99 (802.91 to 833.36)

*Geometric means and 95% CIs were used for biomarkers that were not normally distributed.

†Among the 931 CHD cases, 651 had an MI, 334 had a coronary artery bypass operation and 205 had a coronary angioplasty (some had more than one diagnosis).

CHD, coronary heart disease; CRP, C reactive protein; HDL-C, high-density lipoprotein cholesterol; IFN-γ, interferon-gamma; IL, interleukin; IL-6R, IL-6 receptor; LDL-C, low-density lipoprotein cholesterol; MI, myocardial infarction; TNF-α, tumour necrosis factor-alpha.

### Correlations between individual cytokines

Among controls, plasma levels of CRP were moderately correlated with plasma levels of IL-6 (r=0.45), but were more weakly correlated with TNF-α (r=0.27) (table 2) and with all of the other cytokines studied. Moreover, there were only weak correlations between each of the cytokines, with the strongest correlations of 0.30 observed for IL-6 and TNF-α and for IFN*-*γ and TNF-α, respectively. However, IL-5 was not correlated with any of the other cytokines measured.

**Table 2:** Correlation matrix of plasma cytokines with each other and with selected other plasma biomarkers in controls

	IL-6	IL-5	IFN-γ	TNF-α	IL-6R	LDL-C	HDL-C	Triglycerides	Cystatin C	Fibrinogen
IL-6										
IL-5	0.10									
IFN-γ	0.22	0.07								
TNF-α	0.30	0.03	0.30							
IL-6R	0.13	−0.01	0.02	0.21						
LDL-C	−0.02	−0.06	0.02	0.12	0.09					
HDL-C	−0.24	−0.09	−0.06	−0.14	−0.04	−0.04				
Triglycerides	0.14	0.00	0.07	0.24	0.13	0.30	−0.51			
Cystatin C	0.22	0.07	0.13	0.44	0.20	0.13	−0.13	0.22		
Fibrinogen	0.30	0.01	0.04	0.26	0.10	0.19	−0.05	0.20	0.25	
CRP	0.45	0.05	0.11	0.27	0.07	0.08	−0.18	0.23	0.25	0.46

Values shown are Spearman correlation coefficients.

CRP, C reactive protein; HDL-C, high-density lipoprotein cholesterol; IFN-γ, interferon-gamma; IL, interleukin; IL-6R, IL-6 receptor; LDL-C, low-density lipoprotein cholesterol; TNF-α, tumour necrosis factor-alpha.

### Associations of individual cytokines with CHD

**Figure 1:**
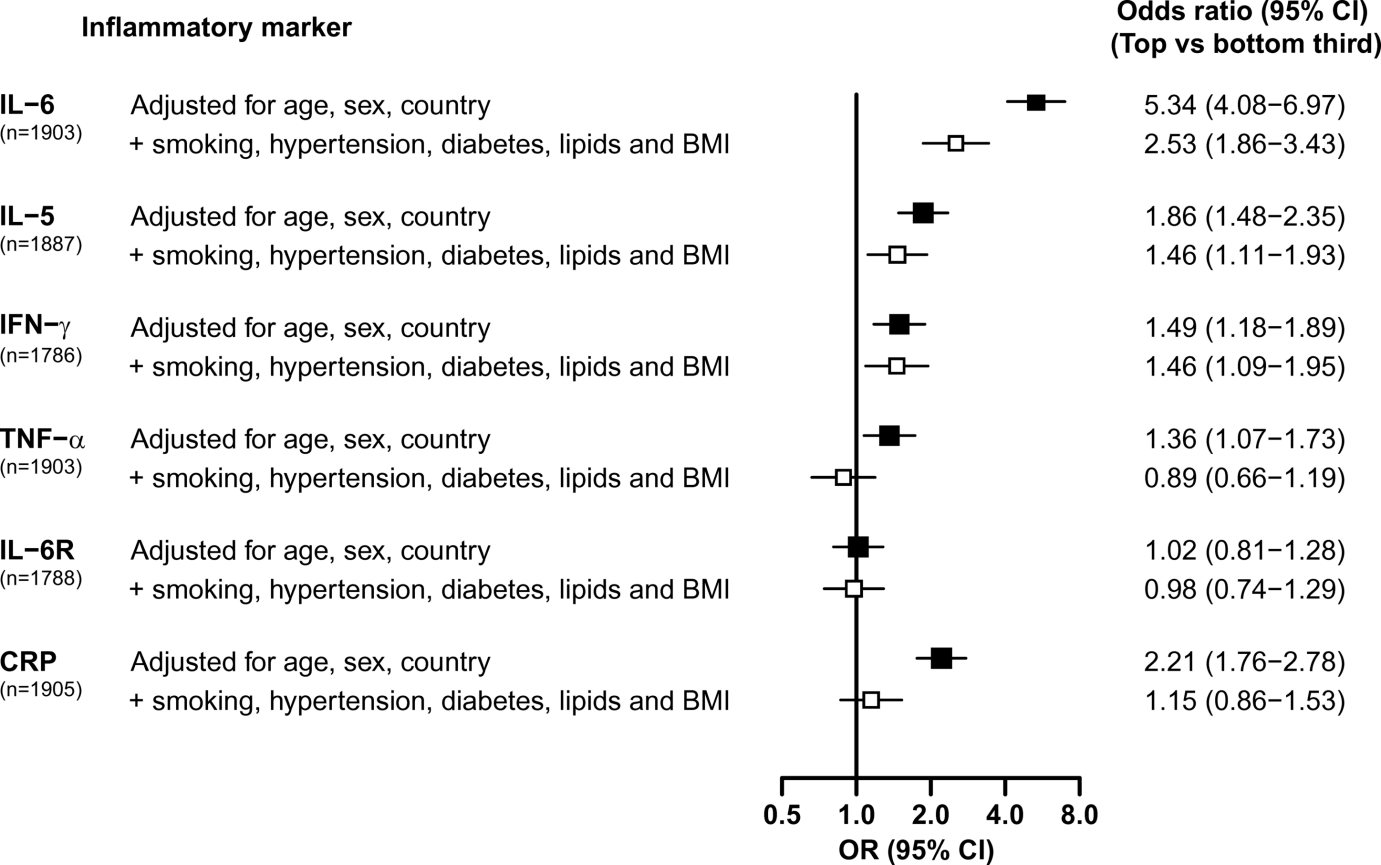
OR (95% CI) for CHD associated with inflammatory markers before and after adjustment for established risk factors. The ORs are the associations of inflammatory markers with CHD in the top relative to the bottom third in controls. The ORs are presented as squares, and the horizontal lines represent the 95% CIs. ORs adjusted for age, sex and country are represented by black squares. The ORs that were additionally adjusted for smoking status, history of hypertension, diabetes status and plasma lipid levels (low-density lipoprotein cholesterol, high-density lipoprotein cholesterol and triglycerides) and body mass index are represented by white squares. BMI, body mass index; CHD, coronary heart disease; CRP, C reactive protein; IFN-γ, interferon-gamma; IL, interleukin; IL-6R, IL-6 receptor; TNF-α, tumour necrosis factor-alpha.

IL-6 was more strongly associated with risk of CHD than any of the other cytokines studied. Individuals in the top versus the bottom third of plasma IL-6 levels had more than a twofold higher risk of CHD (OR 2.53; 95% CI 1.86 to 3.43; p<0.001) after adjustment for established CHD risk factors (figure 1). IL-5 and IFN-γ were also significantly associated with CHD after adjustment for established CHD risk factors, with an approximately 50% higher risk of CHD for individuals in the top versus the bottom third for IL-5 (OR 1.46; 95% CI 1.11 to 1.93; p<0.001) and IFN-γ (OR 1.46; 95% CI 1.09 to 1.95; p=0.01) levels, respectively.

By contrast, neither TNF-α nor IL-6R was significantly associated with CHD before or after adjustment for CHD risk factors. CRP was significantly associated with CHD prior to adjustment for established risk factors, but this effect was completely attenuated and was no longer significant after adjustment for established CHD risk factors (figure 1).

### Independent relevance of individual cytokines

In analyses restricted to the 1769 individuals (93% of total) with complete data on all cytokines significantly associated with CHD after adjustment for established risk factors (ie, IL-6, IL-5 and IFN-γ), the relative independence of the associations of these cytokines from each other was also explored. The association with CHD for individuals in the top compared with the bottom third of IL-5 after adjustment for established CHD risk factors (OR 1.44; 95% CI 1.08 to 1.91; p=0.01) remained significant after additional adjustment for IL-6 (OR 1.36; 95% CI 1.02 to 1.82; p=0.04). However, the association of IFN-γ with risk of CHD was attenuated and no longer significant after additional adjustment for IL-6 (OR 1.29; 95% CI 0.96 to 1.75). The association of IL6 with CHD was not materially altered by adjustment for IL5 or for IFN-γ, as was also the case for associations with IL5 adjusted for IFN-γ.

### Impact of concomitant cardiovascular medication

**Figure 2:**
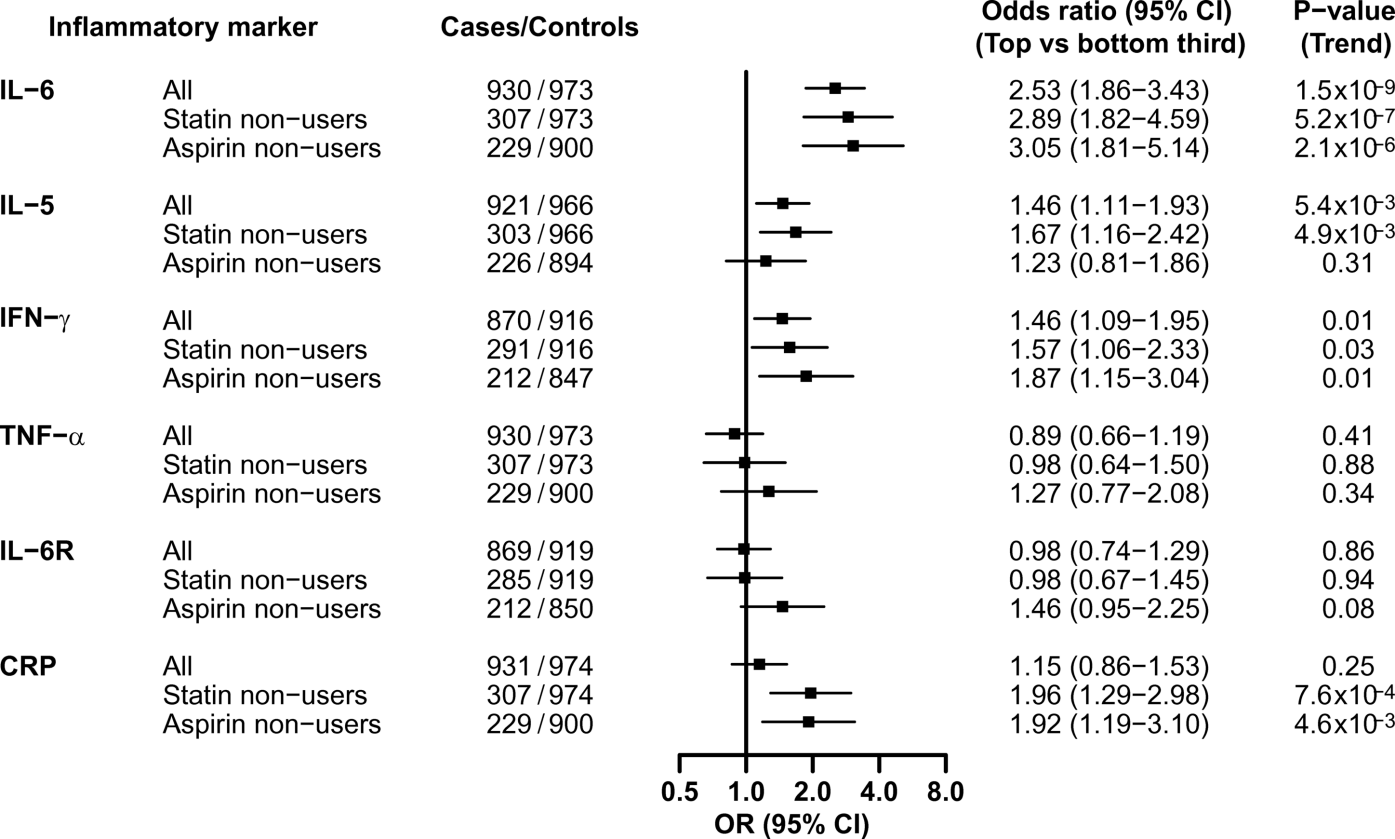
OR (95% CI) for CHD associated with inflammatory markers in all participants and in subsets of non-users of statins or non-users of aspirin therapy. The ORs are the associations of inflammatory markers with CHD in the top relative to the bottom third in controls in all participants and in subsets who were non-users of statins or non-users of aspirin therapy. All analyses were adjusted for age, sex, country, smoking status, history of hypertension, diabetes status and plasma lipid levels and body mass index. Symbols and conventions as in figure 1. CHD, coronary heart disease; CRP, C reactive protein; IFN-γ, interferon-gamma; IL, interleukin; IL-6R, IL-6 receptor; TNF-α, tumour necrosis factor-alpha.

Figure 2 shows exploratory subgroup analyses with the OR of CHD for the top versus the bottom third of IL-6, IL-5, IFN-γ, TNF-α, IL-6R and CRP in all participants and in the subsets of participants who were non-users of statins or non-users of aspirin therapy at the time of recruitment. In each of the subgroups considered (table 1), the effects of individual cytokines on CHD varied (some were weaker and others were stronger), but were generally comparable with the overall results (figure 2).

### Sensitivity analyses

After restricting analyses to a subset in which duplicate cytokine measures were both within range (CV <20% and values within the measurable range for each assay), complete data were available for 1843 (97%) individuals for IL-6, 1536 (82%) for IFN-γ, but only 801 (42%) for IL-5. In this subset, the associations of CHD with both IL-6 and IFN-γ were unaltered (data not shown). While the effect size of IL-5 with CHD was also unaltered (OR 1.46; 95% CI 1.11 to 1.93 versus OR 1.47; 95% CI 0.95 to 2.28 overall versus subset, respectively), the association was no longer significant.

## Discussion

This retrospective case–control study, involving 931 cases and 974 controls, confirmed the importance of IL-6 as the most strongly associated cytokine with risk of CHD, but also demonstrated novel and independent associations of IL-5 with CHD. However, the associations of IFN-γ with CHD were no longer significant after adjustment for IL-6. The findings for IL-6 are consistent with those of a previous meta-analysis of 17 prospective studies which reported OR (95% CI) of CHD of 1.61 (95% CI 1.42 to 1.83) per 2 SD increase in baseline IL-6.^15^ However, the latter study reported that the year-to-year variability of IL-6 values within individuals was relatively high (regression dilution ratios of 0.41; 95% CI 0.28 to 0.53, over 4 years, and 0.35; 95% CI 0.23 to 0.48, over 12 years) and, hence, the OR (95% CI) of CHD after correction for regression dilution bias was 3.34 (2.45 to 4.56).^15^

MR studies have demonstrated the causal relevance of IL-6 for risk of CHD,^4^ and the CANTOS trial has confirmed the reversibility of this excess risk by inhibition of IL-1β that results in lower plasma levels of IL-6 for prevention of CHD.^6^ While genetic variants encoding IL-6R (resulting in increased plasma levels of IL-6) increase risk of CHD, there was no evidence that direct plasma measurements of IL-6R were linked with CHD in the present study.

The findings for IL-5 provide support for a potential role of IL-5 in the aetiology of CHD that warrants further research in large-scale epidemiological studies. The role of IL-5 for risk of CHD was first suggested by IBC 50K CAD Consortium,^7^ but was not confirmed as being genome-wide significant by the CARDIoGRAMplusC4D Consortium.^8^ However, the most recent meta-analysis of CARDIoGRAMplusC4D, involving 60 801 CHD cases and 123 504 controls, reported an OR (95% CI) of CHD for the genetic variant for IL-5 (rs2706399) with each additional copy of the A allele (54% frequency) having an OR of 0.97 (0.96 to 0.99), p=0.007.^8^ Further studies are required to replicate the associations of variants for IL-5 with plasma levels of IL-5 as well as IL-5 genetic variants with risk of CHD. Moreover, eosinophils that include IL-5 receptors have been identified in thrombi, providing biological plausibility of these associations with risk of CHD.^16^ Monoclonal antibodies, such as benralizumab that target IL-5 receptor and lower plasma levels of IL-5, have been shown to be effective for the relief of symptomatic eosinophilic asthma^17 18^ and prompted interest in exploring the potential relevance of this pathway for CHD.

IFN-γ is a proinflammatory mediator that is believed to be the body’s main activator of monocytes and macrophages and more potent than any of the other interferons.^3^ Moreover, IFN-γ is also found in high levels in atherosclerotic plaques.^3^ While the 9p21 CHD susceptibility locus has also been linked with IFN-γ signalling, there are no known inhibitors for IFN-γ. A previous retrospective study of 150 cases and 209 controls also reported associations of IFN-γ with risk of CHD, but did not address the independence of the other cytokines.^18^ Some studies suggested that IFN-γ may control the ratio of matrix metalloproteins and their inhibitors.^19^ Other studies have demonstrated a synergistic effect of both IFN-γ and IL-17 on vascular smooth muscle cells.^20^ However, the present study demonstrated that the association of IFN-γ with risk of CHD was no longer significant after adjustment for IL-6, consistent with what is known about the biological pathway of IL-6 and risk of CHD.^1 3^ Despite this, while trials aimed at inhibiting TNF-α have not shown any beneficial effects on CHD, there is emerging evidence that IFN-γ could be a possible target for prevention of heart failure.^21 22^ MR studies involving much larger numbers of CHD cases are required to integrate the results of both genomic and plasma levels of IFN-γ to assess their causal relevance for heart failure, CHD and other vascular diseases.

The use of a retrospective study design is an efficient and cost-effective approach to assess the strength of association of plasma biomarker concentrations with risk of CHD. However, as with all retrospective studies, it was not possible in the present study to exclude the potential effects of reverse causality on the associations of such biomarkers with disease outcomes. In addition, this study design cannot reliably address the effects of medication on the associations of cytokines with CHD. Moreover, a further limitation of this report was the long interval of 5–10 years between the diagnosis of CHD and blood collection for measurement of cytokines in the cases. However, prospective studies have demonstrated high levels of reproducibility of IL-6 after 4 and 12 years of blood collection versus baseline in control populations, indicating reliable long-term predictive values of such measurements.^15^ Moreover, significant associations of blood lipids^23 24^ or other biomarkers^25^ with CHD reported in previous retrospective studies have been subsequently corroborated in prospective studies,^25 26^ although the strength of such associations in prospective studies is typically less extreme than in retrospective studies.^23–26^ Hence, retrospective studies are useful for discovery, and any significant positive associations in such studies can be replicated and confirmed in prospective studies. While the cytokine assays studied had high interassay CV (particularly for IFN-γ), all cytokine assays were measured in duplicate for the present report.

In conclusion, the findings of this study demonstrating independent associations of both IL-6 and IL-5 with risk of CHD provide support for further large-scale investigations of these cytokines. This study also highlights the need for further investigation using larger panels of cytokines in studies of CHD risk, which could enhance our understanding of the biology of CHD and also lead to the discovery of novel treatments.^22 27–29^
